# Hospital Meetings, &c.

**Published:** 1897-05-22

**Authors:** 


### HOSPITAL MEETINGS, &C.

### THE MARY WARDELL CONVALESCENT HOME FOR SCARLET FEVER.

A meeting on behalf of Miss Mary Wardell's Convalescent Home for scarlet fever patients was held at Grosvenor House on Monday, May 17th. The Duke of Westminster had hoped to take the chair, but being unavoidably prevented his place was filled by the Earl of Derby. Letters regretting inability to attend were received from the Duchess of Albany, Lord Lister, Dr. Thorne Thorne, and others. Amongst those present were Sir R. Douglas Powell, Bart., M.D., the Archdeacon of London, Miss Wardell, and Dr. Burnet, chairman of the committee.

The objects of the meeting were specially to give some account to the public of the work done during the past twelve years, since the last meeting at the Mansion House; to raise the sum of £2,000 to clear the home of all debt, and provide for the thorough cleaning, painting, and general repairs which are needed; and to increase the number of annual subscriptions so as to meet the necessarily heavy expenses of the home and extend its usefulness. It is now more than twelve years since the home was opened by the Prince and Princess of Wales, and since that time over 2,600 patients have been admitted. The home has thus conferred the greatest possible benefit on numbers of sufferers from scarlet fever. Two classes of patients are admitted to the home. It was at first intended for the reception of the working classes only, paying but a fraction of their cost to the institution, but in response to earnest requests for an extension of its benefits to people able to pay on a higher scale, a certain number of "first-class" patients are also taken in.

The cost of maintenance is necessarily high in a home of this description, for many are the expenses, and as the home cannot be self-supporting if it is to help the poor, additional subscriptions and donations are most urgently needed if its useful work is to be maintained. Miss Wardell has unsparingly devoted her time and money to carry out her cherished scheme, and has earned the gratitude of thousands who have reaped the benefit of her unselfishness. The difficulty of keeping up the funds to the required level is now pressing heavily upon the foundress, and the public are urgently appealed to for their support, and by extending a knowledge of the home and its beneficent work amongst others to contribute towards its continued usefulness.

### KING'S COLLEGE HOSPITAL.

Among those present at the annual festival dinner in aid of King's College Hospital, which was held at the Whitehall Rooms on Friday, the 14th inst., were Lord Glenesk (in the chair), Viscount Dillon (who has recently accepted the post of chairman of the Committee of Management), Lord Davey, the Hon. F. W. D. Smith, M.P., Mr. Richard Twining (treasurer), the Rev. Dr. Wace, Sir Alfred C. Lyall, General Moberley, General Sim, Canon Elwyn, Canon Benham, Dr. Curnow, Sir Hugh Beevor, Bart., and Dr. W. S. Playfair. The Chairman, in proposing the toast of the evening, "Success to King's College Hospital," said that the hospital had fallen on good times. The report was a most satisfactory one, both because the hospital had been successful in obtaining money, and because, at the same time, great and important economies had been effected. The Rev. Dr. Wace, in responding, after briefly reviewing the improvements effected during the ten years he had been chairman of the Committee of Management, said that he was glad to hear from the warden that the Prince of Wales's fund had not, so far as he could see, interfered to any extent with the contributions from the friends of the hospital. The amount collected by means of the dinner was £2,760.

### CHELSEA HOSPITAL.

The annual meeting of the governors of the Chelsea Hospital for Women was held on Thursday, the 13th inst., at the hospital, Lord Glenesk presiding. The report for the past year showed that the finances of the hospital at the end of 1896 were in a satisfactory condition, but that this was due in great measure to the collection of a special fund of £1,930 for announcement at the previous annual meeting in honour of the twenty-fifth anniversary of the charity's existence. Unless, therefore, some substantial help of a similar nature were forthcoming during the present year, it was feared that a heavy debt would have to be incurred at the bankers. The loss of annual subscriptions through the death of many supporters had been severely felt. The results in the treatment of patients were even better than in 1895, the rate of mortality being but 1·4. The Convalescent Home was proving an invaluable adjunct to the hospital but needed increased support. Lord Glenesk, in moving the adoption of the report, further stated that the statistics for 1897 more than equalled those just quoted. There had been more patients, and a much greater number of severe cases, but the rate of mortality had not increased. Economy had been effected in many departments, though not to the detriment of the patients, diet, or treatment, and a comparison with other hospitals of the same class would prove very favourable to the Chelsea Hospital for Women.

### A POPLAR MEETING.

The latest association of faddists—if the word sufficiently indicates persons who want to have a finger in everything that they know nothing about—has bestowed on itself the title of "The Society for the Protection of Hospital Patients," and has secured the services of Miss Beatty (of Beatty v. Cullingworth fame) as honorary secretary.

This society convened a public meeting in the Poplar Town Hall on Friday, the 14th, to protest against "the frequent performance of experiments upon human subjects in metropolitan hospitals," and "against the handing over of grants from the Prince of Wales's Fund to hospital authorities until some guarantee be given that the patients would not be used for purposes of research to their detriment." There was a fair attendance of working men. The proceedings were distinctly animated, and there were frequent interruptions, but considering the wild and contradictory statements made by the platform speakers, they were given a remarkably fair hearing. Of these statements, one, that "the metropolitan hospitals spend fifty per cent. of their income on advertisements," may be taken as a fair sample.

The Hon. Sydney Holland made a vigorous reply on behalf of himself and the Poplar and London Hospitals, and proposed the following amendment: "That those present have heard with extreme regret the statements and charges made against the London hospitals and do not believe a word of them." This was seconded by a dock labourer, and carried, there being but three dissentients in the body of the hall, and the meeting broke up with three vigorous cheers for Mr. Holland and the Poplar Hospital.

